# Gestational Diabetes Triggers Oxidative Stress in Hippocampus and Cerebral Cortex and Cognitive Behavior Modifications in Rat Offspring: Age- and Sex-Dependent Effects

**DOI:** 10.3390/nu12020376

**Published:** 2020-01-31

**Authors:** Maribel Huerta-Cervantes, Donovan J. Peña-Montes, Rocío Montoya-Pérez, Xóchitl Trujillo, Miguel Huerta, Miguel Ángel López-Vázquez, María Esther Olvera-Cortés, Alfredo Saavedra-Molina

**Affiliations:** 1 Instituto de Investigaciones Químico-Biológicas, Universidad Michoacana de San Nicolás de Hidalgo, 58030 Morelia, Mich., Mexico; marzy112@yahoo.com.mx (M.H.-C.); yodonnie@gmail.com (D.J.P.-M.); rmontoya@umich.mx (R.M.-P.); 2 Centro Universitario de Investigaciones Biomédicas, Universidad de Colima, 28045 Colima, Colima, Mexico; rosio@ucol.mx (X.T.); huertam@ucol.mx (M.H.); 3 Centro de Investigación Biomédica de Michoacán, Instituto Mexicano del Seguro Social, 58341 Morelia, Mich., Mexico; migangelv@yahoo.com.mx

**Keywords:** anxiety, behavior, cortex, gestational diabetes, hippocampus, learning, metabolism, oxidative stress

## Abstract

Gestational diabetes (GD) has been linked with an increased risk of developing metabolic disorders and behavioral abnormalities in the offspring. Oxidative stress is strongly associated with neurodegeneration and cognitive disruption. In the offspring brains in a GD experimental rat model, increased oxidative stress in the prenatal and postnatal stages was reported. However, long-term alterations to offspring behavior and oxidative stress, caused by changes in the cerebral cortex and hippocampus, remain unclear. In this study, we evaluated the effect of GD on young and adult male and female rat offspring in metabolic parameters, cognitive behavior, and oxidative stress. GD was induced using streptozotocin in dams. Next, the offspring were evaluated at two and six months of age. Anxiety-like behavior was evaluated using the elevated plus maze and open field maze; spatial learning and short-term memory were evaluated using the Morris water maze and radial maze, respectively. We determined oxidative stress biomarkers (reactive oxygen species (ROS), lipid peroxidation and glutathione status) and antioxidant enzymes (superoxide dismutase and catalase) in the brain of offspring. We observed that male GD offspring showed a reduced level of anxiety at both ages as they spent less time in the closed arms of the elevated plus maze at adult age ((*P* = 0.019, *d* = 1.083 ( size effect)) and spent more time in the open area of an open field (*P* = 0.0412, *d* = 0.743) when young and adult age (*P* = 0.018, *d* = 0.65). Adult female GD offspring showed a reduced level of anxiety (*P* = 0.036; *d* = 0.966), and young female GD offspring showed a deficiency in spatial learning (*P* = 0.0291 vs. control, *d* = 3.207). Adult male GD offspring showed a deficiency in short-term memory (*P* = 0.017, *d* = 1.795). We found an increase in ROS and lipid peroxidation, a disruption in the glutathione status, and decreased activity of catalase and superoxide dismutase (*P* < 0.05 vs. control, *d* > 1.0), in the cerebral cortex and hippocampus of male and female GD offspring. GD altered metabolism; male offspring of both ages and adult females showed a high level of triglycerides and a lower level of high-density lipoprotein-cholesterol (*P* < 0.05 vs. control, *d* > 1.0). Young and adult female offspring displayed higher insulin levels (*P* < 0.05, *d* > 1.0). These results suggest that gestational diabetes modifies oxidative stress and cognitive behavior in an age- and sex-dependent manner.

## 1. Introduction

Gestational diabetes (GD) is a temporary disorder of glucose metabolism during pregnancy, is diagnosed with a fasting glucose level >126 mg/dL, 2-hour glucose 200 mg/dL following a 75 g oral glucose or a casual glucose >200 mg/dL [1]. Currently, one out of six human births are affected by gestational diabetes. Of live births, 21.3 million or 16.2% had some form of hyperglycemia in pregnancy diabetes [2] GD affects offspring at different critical developmental periods during the fetal stages and early postnatal life, and these alterations enhance the risk of health disturbances in adulthood, like metabolic syndrome, diabetes, and obesity [3,4,5,6].

In animal models, GD was shown to cause alterations in the neonatal body weight of offspring, as well as alterations in the fetal pancreatic function, morphology, number and size of pancreatic islets, resulting in glucose intolerance or insulin resistance in different organs, such as liver, muscle, and adipose tissue [7,8,9]. Whereas the effect of GD has been studied in peripheral organs, the effects of GD on the behavior and neural development in the cerebral cortex and hippocampus remains unclear. The hippocampus (the hippocampal formation includes Cornu Amonis (CA) 1–4, dentate gyrus, entorhinal cortex, subiculum, and pre- and para-subiculum) is a particularly important structure for processing spatial learning and memory, and together with the cerebral prefrontal cortex, participates in working memory and executive function organization. The development delay and/or damages to these structures are responsible for cognitive deficiency [10]. Some studies in humans have described that maternal diabetes affects the intrauterine development of the nervous system and cognitive abilities of the offspring. While the offspring of mothers with gestational diabetes show alterations in prenatal neurodevelopment, explicit memory [11], as well as electrophysiological alterations related attention deficit [12], and motor alterations [13].

Studies on diabetic rat offspring have reported retarded dendritic development and lower expression of the insulin-like growth factor at the embryonic age [14], a critical factor in neuron growth, dendritic arborization and synaptogenesis [15]. A significant reduction in the pyramidal cell density of CA1 and CA3 hippocampal subfields at postnatal days 7 and 21 was reported in GD offspring [16]. 

Several studies have indicated that rats with GD and their offspring are exposed to oxidative stress, reporting increased biomarkers of oxidative stress in the liver, muscle, pancreatic cells, and brain [17,18]. In the brain, oxidative stress is involved in the neuroinflammatory state and excitotoxicity, two main causes of neural death [19]. In humans and experimental models, oxidative stress has been linked to cognitive impairment in neurodegenerative diseases, like Alzheimer’s and Parkinson’s, as well as in age-related cognitive decline [20]. Neurons are particularly vulnerable to oxidative damage due to their high polyunsaturated fatty acid composition in membranes and their high oxygen and energy demand and glucose consumption [21]. Therefore, determining the role of oxidative stress in GD offspring rats is necessary. 

Cognitive impairment in the diabetic milieu has been amply recognized in diabetes mellitus (DM) 1 and 2 [22,23]. In rats, feeding a diet high in sucrose or fat was shown to increase anxiety-like behavior [24,25] but the relationship between hyperglycemia in the intrauterine milieu, as in the case of gestational diabetes (GD), and its impact in the cognitive development and behavior of offspring and a possible mechanism has been poorly studied. The aim of this study was to evaluate the effects of gestational diabetes on cognitive performance, focusing on anxiety-like behavior and learning in juvenile and adult offspring rats. We also investigated the impact of gestational diabetes on oxidative stress and metabolic parameters in the hippocampus and cerebral cortex at the same ages.

## 2. Materials and Methods 

### 2.1. Experimental Design

In this study we employed a total of twelve Wistar female rats, weighing 280–300 g. Rats were maintained under a normal light/dark cycle (12/12 h) at a temperature of 22 °C in an animal house, with free access to standard rat food and water (Rat Diet 5012, Lab Diet, St Louis, MO, USA). All experiments were conducted in accordance with the National Institutes of Health Guide for the Care and Use of Laboratory Animals (NIH Publication No.80-23) and the “Norma Oficial Mexicana” regarding the use of experimental animals (NOM-062-ZOO-1999), and approved by the Research Ethics Committee of the Instituto Mexicano del Seguro Social (IMSS) (R-2017-1603-16).

Female rats were mated with male control rats. Next, vaginal smears were performed over consecutive days to verify the presence of sperm. Once sperm was observed, gestational day 0 had begun, and pregnant rats (dams) were randomly divided into two groups: gestational control rats (GC, *n* = 6) and gestational diabetic rats (GD, *n* = 6). On the ninth day of gestation (mid gestation), diabetes was induced, after 16 h of fasting, by a single intraperitoneal injection of streptozotocin (STZ, 38 mg/kg of body weight), freshly dissolved in citrate buffer (0.1 M, pH 4.5), and GC rats were injected with an equivalent volume of a citrate buffer. To select the STZ dose used in this study we carry out a screening injecting the drug (STZ) at different doses (35 to 45 mg/kg, intraperitoneally). A 38 mg/kg dose was selected taking into account that elevated glucose levels were maintained in a sustained manner and keeping the pregnancy viable and the viability of fetuses, as well as their development. Gestational diabetes was confirmed by a tail blood sample, and the glucose level was measured using a glucometer (Accu-chek Performa System, Roche Diagnostics GmbH, Mannheim, Germany) two days later. Only rats that exhibited blood glucose levels between 200 and 350 mg/dL were considered in this study. Glucose levels were monitored throughout pregnancy by taking postprandial blood on days 12, 16, and 20 of pregnancy. At born, all litters were adjusted to 12 pups, maintaining an equal number of males and females. We used two male and two female rats from each litter; after weaning, rats were maintained in groups of four per cage, until the behavioral evaluation. Two-month-old rats (young) and six-month-old rats (adult) male and female offspring from the gestational control (GC) and gestational diabetes (GD) dams; were studied. Behavior tests were applied to 10–12 rats from each group. Next, 5–6 rats were sacrificed to determine oxidative stress biomarkers and the other half of the rats was perfused with formaldehyde solution and reserved for subsequent morphological studies.

### 2.2. Behavioral Test

Anxiety-like behavior was evaluated using the elevated plus maze and open field test. The Morris water maze was used to evaluate spatial learning, and the eight-arm radial maze was employed to assess spatial working memory. 

#### 2.2.1. Elevated Plus Maze

First, anxiety-like behavior was evaluated by employing the elevated plus maze. The maze consisted of two opposite open arms (50 × 10 cm) and two opposite enclosed arms (50 × 10 × 40 cm); with an open roof. The maze was elevated to a height of 50 cm and lighted by a 60-watt white lamp in the center of the maze. Rats were individually placed in the central area of the maze, and their behavior was recorded for 10 min and analyzed using the Sci-Works software (DataWave Technologies Inc., Longmont, CO, USA). The time spent in the closed arms was measured.

#### 2.2.2. Open Field Test

We also evaluated anxiety-like behavior using the open field test. The test consisted of a square arena (60 × 60 cm), divided into eight quadrants (peripheral areas, including corner areas) and a central zone. The arena was lighted by a 60-watt white lamp, situated in the center. Similar to the elevated plus maze, the rats were placed individually into the central area, and their behavior was recorded for 10 min and analyzed using the Sci-Works software (DataWave Technologies Inc., Longmont, CO, USA). The total time spent in the open area (central zone) was measured.

#### 2.2.3. Morris Water Maze

Next, spatial learning was evaluated using the Morris Water Maze. The maze consisted of a circular pool (measuring 100 cm diameter and 28 cm high, for young rats, 150 cm diameter and 50 cm high, for adult rats) filled with water, maintained at 27 ± 2 °C, and dyed dark blue by the addition of gentian violet. The pool contained a circular platform that was placed 2 cm under the water level in a fixed position in one of the four virtual quadrants of the maze during the study. Visual cues were located around the experimental room. Ten different starting positions were placed around the perimeter of the pool. The trial began when a rat was placed in the water facing the wall of the pool at one of the starting points. If the rat failed to find the platform within 60 seconds, it was guided to the platform by the experimenter. Once the rat reached the platform, it was allowed to remain there for 15 seconds.

Training consisted of two trials per day (beginning from 1 of the 10 starting positions, which was chosen randomly, with inter-trial period of 20 minutes for 8 consecutive days. On the ninth day, after the last training session, spatial memory was evaluated. Finally, rats were challenged by removing the platform from the pool and allowing them to swim for 30 seconds (probe trial). The swimming routes were recorded and analyzed using the Sci-Works software (DataWave Technologies Inc., Longmont, CO, USA). The escape latency and distance travelled during the 8 days of training, as well as the time spent at each quadrant during the probe trial, were measured.

#### 2.2.4. Radial Maze

The eight-arm radial maze was employed to evaluate short-term memory. The maze consisted of one central circular arena (30 cm in diameter), from which eight radial arms were extended (60 cm × 10 cm). For this behavioral test, rats were maintained under a food restriction condition (80% of their normal consumption ad libitum) for two weeks, followed by a habituation period: Rats were familiarized with the maze during two sessions. On the first day, they were maintained in the maze for 10 minutes, where free exploration occurred; on the second habituation day, rats were exposed again to the maze for 10 min, with a reward (fruit cereal) available in the central area and arms of the maze. On the third day, the training period, the rats were placed in the central area with the eight available arms. The number of visited arms was recorded, and a rat travelling with four feet into the arm was considered a visit. The visited arms were recorded during a maximal period of 10 min, or until the rat had eaten in all arms. Rats performed two 20-minute trials per day over eight consecutive days. Re-entry errors (entries to a previously visited arm), omission errors (entries to the arm, without collection of the reward), and total errors (re-entry errors plus omission errors) were evaluated.

Once the behavioral tests were accomplished, rats were sacrificed by decapitation. The brain was dissected, and blood samples were taken.

### 2.3. Tissue Preparation

The bilateral hippocampus and cerebral cortex were dissected. Next, tissues were gently chopped. Then, a part of the tissues was homogenized with a buffer (70 mM sucrose, 20 mM mannitol, 1 mM ethylene glycol tetraacetic acid (EGTA), 0.5 % bovine serum albumin (BSA) and 10 mM 3-(N-Morpholino) propanesulfonic acid (MOPS), pH 7.4) to assay the oxidative stress biomarkers (glutathione, reactive oxygen species (ROS), superoxide dismutase (SOD), and catalase activities). The other part was homogenized in 0.9% saline (sodium chloride) and employed for the determination of lipid-peroxidation. Homogenates were stored at −70 °C, until use. The protein content of the homogenates from the cerebral cortex and hippocampus was assayed by a modification of the Biuret procedure [26] using BSA as the standard.

### 2.4. Oxidative Stress Biomarkers

#### 2.4.1. Determination of Lipid Peroxidation 

Lipid peroxidation was measured by thiobarbituric acid-reactive substances (TBARS) as described by Buege and Aust [27] with slight modifications. Next, 500 µg of protein was suspended in phosphate buffer (100 mM, pH 7.4), mixed with the reagent solution containing 0.375% thiobarbituric acid (TBA), 15% trichloroacetic acid (TCA), and 0.25 M hydrochloric acid (HCl), and incubated for 15 min in a boiling water bath. Butylated hydroxytoluene (BHT) 0.01% was added to the TBA-TCA-HCl reagent to prevent non-specific chromophore formation. After cooling, the flocculent precipitate was centrifuged at 6720 ×g for 5 min. The absorbance was measured at 532 nm in a Perkin Elmer Lambda 18 UV VIS Spectrophotometer (Perkin Elmer Inc., Shelton, CT, USA) using 156 mM−1 cm−1 as the molar extinction coefficient.

#### 2.4.2. Determination of ROS 

ROS were determined using the cell-permeable fluorescent probe 2',7'-dichlorodihydrofluorescein diacetate (H2DCFDA). We resuspended 500 µg of protein from the different tissues in 2 mL of buffer (10 mM 4-(2-hydroxyethyl)-1-piperazineethanesulfonic acid (HEPES), 100 mM KCl, 3 mM MgCl_2_, and 3 mM KH_2_PO_4_, pH 7.4) and incubated with 12.5 µM of H_2_DCFDA for 15 min in an ice bath under constant shaking [28]. Next, basal fluorescence was recorded after 1 min, and 5 mM/5mM of glutamate/malate was added. Changes in fluorescence were recorded for 20 min at extinction 485 nm and emission 520 nm wavelengths, in a spectrofluorophotometer (Shimadzu RF-5301PC, Kyoto, Japan). Results are expressed as arbitrary units. 

#### 2.4.3. Determination of Glutathione Status

Glutathione status was determined in accordance with Akerboom and Sies [29]. The total glutathione was determined in 500 µg of protein. Samples were suspended in 0.1% Triton-X and 0.6% sulfosalicylic acid in a 0.1 M potassium phosphate buffer, plus 5 mM ethylenediaminetetraacetic acid (EDTA), pH 7.5. Next, the mix was sonicated in three cycles of sonication/ice for 20 s, followed by two freeze/defrost cycles, and centrifuged at 6500 ×g. Then, the supernatant (100 μL) was placed in potassium phosphate buffer, with 100 µM 5,5′-Dithiobis (2-nitrobenzoic acid) (DTNB) and 0.1 units/mL glutathione reductase (GR) and incubated for 30 seconds. The reaction was started by the addition of 50 µM β-NADPH and monitored for 5 min at 412 nm in kinetic mode in a spectrophotometer UV VIS (Shimadzu RF-5301PC, Kyoto, Japan). Oxidized glutathione (GSSG) was obtained after reduced glutathione (GSH) derivatization by incubation with 0.2% 4-vinylpyridine for 1 h at room temperature, before the reaction. GSH was calculated by subtracting GSSG from the total glutathione. 

#### 2.4.4. Determination of Catalase and SOD activities

Catalase activity was assayed by measuring the conversion of H_2_O_2_ to oxygen using a Clark-type oxygen electrode connected to a biological oxygen monitor (5300A Biological Oxygen Monitor, YSI, Yellows Springs, OH, USA), according to Jeulin et al. [30], with slight modifications. Briefly, 500 µg of protein from homogenate tissues were resuspended in a 0.1 M potassium phosphate buffer, with 5 mM EDTA (pH 7.6), at 25 °C and monitored 1 minute. Later, fresh 6 mM of H_2_O_2_ was added to the chamber, and the conversion of H_2_O_2_ to oxygen was recorded for 2 minutes. Finally, 1.0 mM of sodium azide was added to the chamber. Catalase activity was calculated using bovine catalase as standard. Results are expressed as U × mg of protein. SOD activity was determined using a commercial kit (Sigma, 19160, St. Louis, MO, USA), following the manufacturer’s instructions, and calculated using the SOD from *Escherichia coli* as standard.

### 2.5. Biochemical Serum Analyses

Blood glucose levels were determined using a commercial blood glucometer (Accu-chek Performa System, Roche Diagnostics GmbH, Mannheim, Germany), serum lipid (triglycerides, total cholesterol and high-density lipoprotein (HDL) levels were determined enzymatically using reagent kits and following the manufacturer instructions (Química Clínica Aplicada, Amposta, Spain). Serum insulin levels were measured by using a sandwich-type immunoassay (ELISA) commercial kit (80-INSRT-E01, E10, ALPCO Diagnostic, Salem, NY, USA). 

### 2.6. Statistical Analysis

All data were analyzed using Prism (GraphPad 7.0 version, Inc., San Diego, CA, USA). Results are expressed as the mean ± standard error of the mean (SEM), and significance of differences in the mean of body weight, metabolic parameters, anxiety-like behavior, and oxidative stress biomarkers were assessed using an unpaired, a two-tailed Student’s *t*-test. The Morris water maze data were analyzed using a two-way analysis of variance (ANOVA) with the group as the between-subject factor and day as the within-subject factor. Tukey post hoc tests were used if the overall differences were significant (*P* < 0.05). An analysis of the effect size (*d*) was conducted if the outcome was statistically significant. Finally, the Pearson correlation coefficients were calculated between behavioral parameters and oxidative stress biomarkers was made.

## 3. Results

### 3.1. Effects of STZ on Pregnant Female Rats (F0)

A total of six female rats (dams) for group (control and GD) were required to obtain the total male offspring and female offspring for each group and age. Glucose fasting was measured two days after the STZ injection, and a significant increase in fasting glucose in GD, compared with GC female rats was observed (*t* = 2.944, *P* = 0.0321). As shown in Table 1, gestational diabetes was characterized by an increase in the postprandial blood glucose. Glucose levels in the GD group were four times greater than in the GC group and remained high throughout pregnancy (*F* (1,10) = 84.12, *P* = 0.0003). We observed as no difference in body weight between rats from the GD and GC groups.

### 3.2. Effects of GD on the Body Weight of First-Generation Offspring

We measured body weight at birth and weaning of all rats in the litter, then all rats were randomly divided into two groups: Young and adult. Male offspring of diabetic rats showed a lower body weight at birth. However, during weaning, female and male offspring of GD rats showed a reduced body weight (33% less in male and 26% less in female), compared with their respective controls. Only young male GD offspring showed a reduced body weight (15%), and the reduction remained into adulthood (Table 2).

### 3.3. Effects of GD on the Anxiety-Like Behavior of First-Generation Offspring

**Figure 1:**
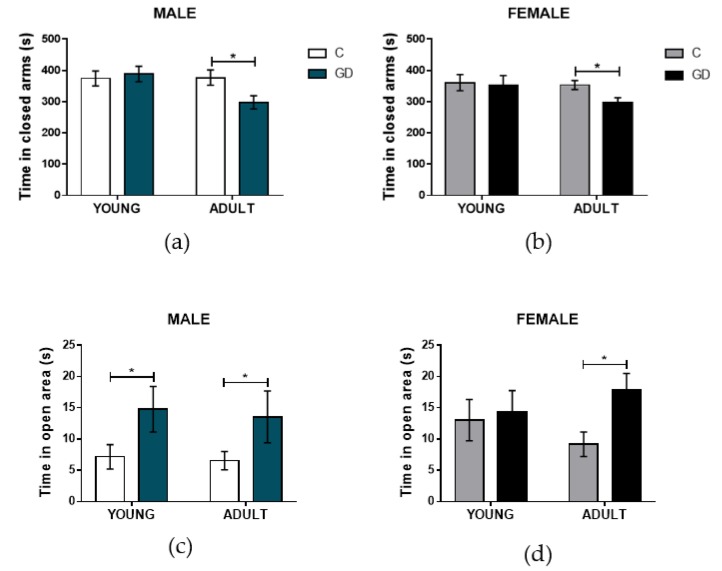
Effect of gestational diabetes (GD) on anxiety-like offspring behavior. Time spent in closed arms of the elevated plus maze for the (**a**) male and (**b**) female offspring rats. Time spent in the open area of the open field for the (**c**) male and (**d**) female offspring rats. C, control offspring; GD, gestational diabetes offspring. Control male offspring: young, *n* = 10; adult, *n* = 12; GD male offspring: young, *n* = 10; adult, *n* = 12. Control female offspring: young, *n* = 10; adult, *n* = 10; GD female offspring: young, *n* = 10; adult, *n* = 10. Data are expressed as the mean ± SEM; Student’s *t*-test; * *p* < 0.05.

To determine the effect of GD on the anxiety of the offspring, the performance of rats in the plus maze and open field were evaluated. In the plus maze, the time that rats spent in the closed arms is considered an anxiety index. Male and female GD offspring spent a similar amount of time in the closed arm, compared with their respective control group when young (Figure 1a, b). Both GD male and female adult rats showed a decrease in the time spent in the closed arms (*t* = 2.42, *P* = 0.0193, *d* = 0.992, and *t* = 2.53, *P* = 0.0199, *d* = 1.083, respectively), suggesting that GD male and female offspring rats experienced a reduced level of anxiety in adulthood compared with the normoglycemic offspring. In the open field, the time spend in the center (open area) is considered an anxiety level index (rats spending more time in this open area are considered less anxious). As shown in Figure 1c,d, young and adult male GD offspring rats spent twice as much time in the center of the open field as the control offspring, whereas the GD offspring female rats spend an 80% more time in the center of the open field only in adulthood. In agreement with the plus maze performance, these results strongly indicate a reduced anxiety level in adult GD offspring, regardless the sex, whereas when young, only GD male offspring showed reduced anxiety levels in the open field test. 

### 3.4. Effects of GD on the Spatial Learning of First-Generation Offspring

**Figure 2:**
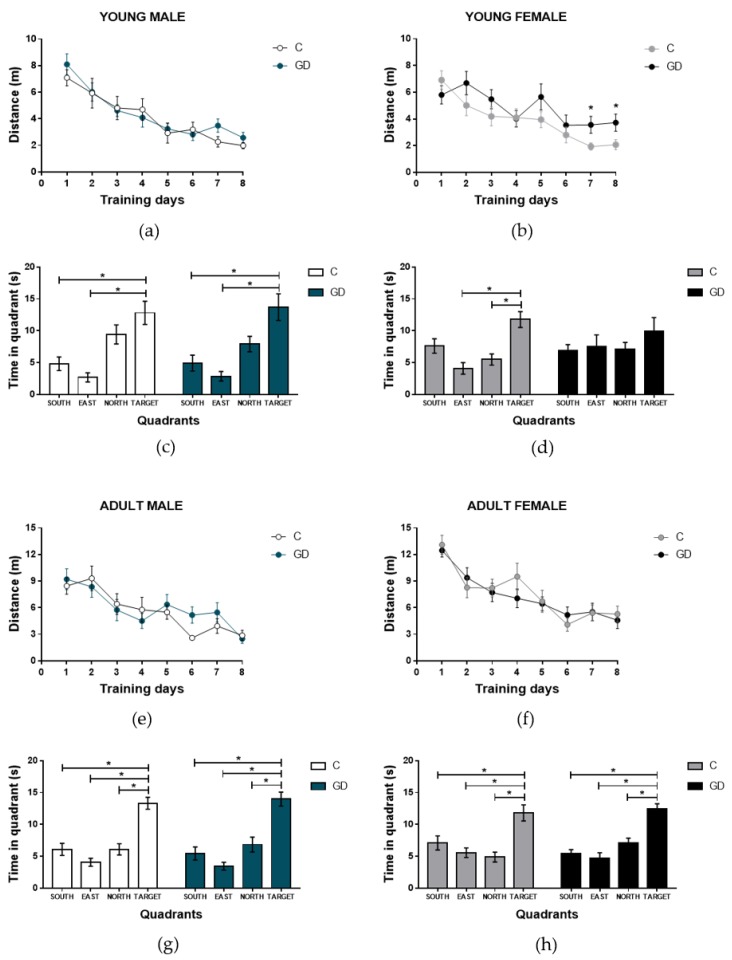
Effect of GD on the spatial learning of offspring, showing the distance traveled (m) during the training days and the probe trial and the time spent at each quadrant. The young male and female offspring distance (**a, b**) and time in each quadrant of young male and female offspring **(c, d**)**.** The adult male and female offspring distance (**e, f**) and time in each quadrant of adult male and female offspring (**g, h**). C, control offspring; GD, gestational diabetes offspring. Control male offspring: young *n* = 10; adult *n* = 12, GD male offspring: young *n* = 10; adult *n*=12. Control female offspring: young, *n* = 10; adult, *n* = 10; GD female offspring: young: *n* = 10; adult, *n* = 10. Data are expressed as the mean ± SEM. Two-way ANOVA of repeated measures and one-way ANOVA; post hoc Tukey’s test; * *p* < 0.05.

To determine if GD disrupts spatial learning, the performance of young and adult (male and female) offspring of GD rats was assessed using the Morris water maze test for eight days. No statistical differences were found in terms of the swimming distances of male rats of any age during the training days compared with the control group (*F* (1,9) = 1.07, *P* = 0.32 and *F* (1,11) = 0.41, *P* = 0.53 for the young and adult age groups, respectively). In the probe trial (ninth day), rats from both groups (GD and C groups) spent more time at the platform position (target) instead of the other maze quadrants, and the performance of the groups was similar, as shown in Figure 2a–d. The young female offspring of GD rats showed longer swimming distances during the training days (group effect, *F* (1,9) = 6.71, *P* = 0.029, *d* = 3.207), and paired comparisons showed significantly longer distances at days 7 and 8 for the GD compared with the female C group (*t* = 2.362, *P* = 0.029, *d* = 1.056 and *t* = 2.216, *P* = 0.039, *d* = 0.991, respectively). Accordingly, with deficient learning, in the probe trial (ninth day), GD female rats spent the same amount of time in all zones of the maze, whereas the rats from the C group searched the platform for the target quadrant, as shown in Figure 2e,f. These changes were not observed in adulthood, as shown in Figure 2g,h.

### 3.5. Effects of GD on the Working Memory of First-Generation Offspring 

**Figure 3:**
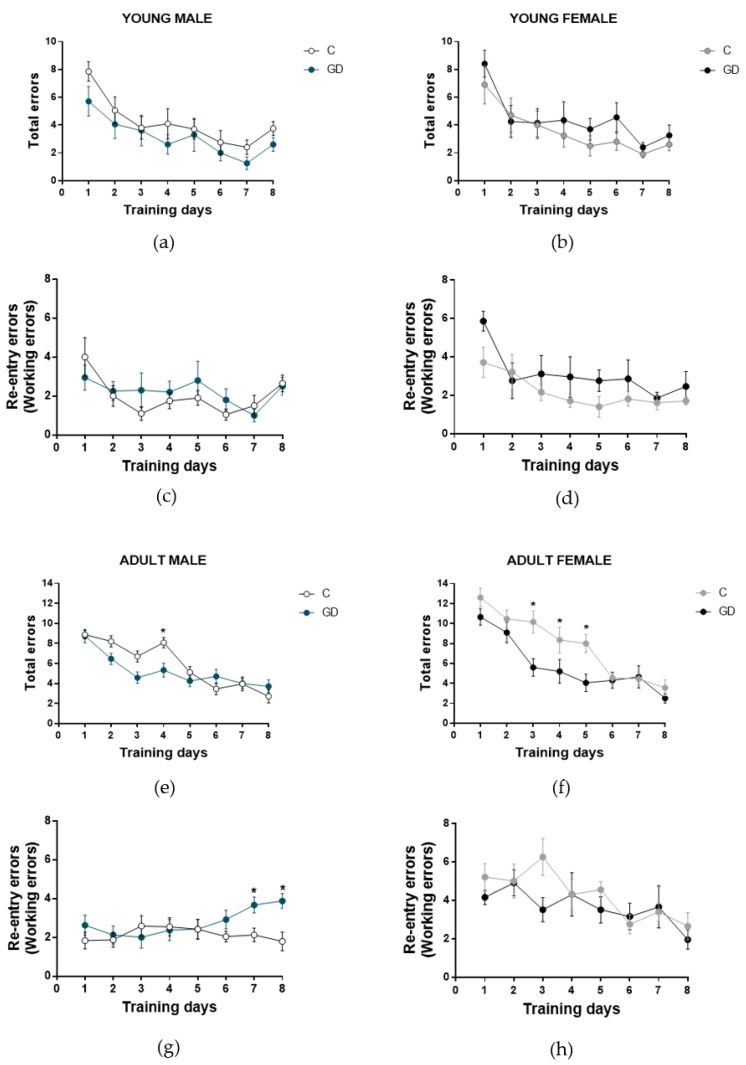
Effect of GD on the short-term memory of offspring, showing the total and reentry errors during the training days. The young male and female offspring total errors (**a**, **b**) and reentry errors (**c**, **d**) respectively. The adult male and female offspring total errors (**e, f**) and reentry errors (**g, h**) respectively. C, control offspring; GD, gestational diabetes offspring. Control male offspring: young, *n* = 10; adult, *n* = 12. GD male offspring: young, *n* = 10; adult, *n* = 12. Control female offspring: young, *n* = 10; adult, *n* = 10. GD female offspring: young, *n* = 10; adult, *n* = 10. Data are expressed as the mean ± SEM. Two-way ANOVA of repeated measures and one-way ANOVA; post hoc Tukey’s test; * *p* < 0.05.

Next, to evaluate the effect of GD on the spatial working memory of offspring, we used an eight-arm, eight-day radial maze test (Figure 3). Young male and female GD offspring did not show statistical differences in the number of total and re-entry errors, compared with the control group (*F* (1,9) = 1.72, *P* = 0.22 and *F* (1,9) = 0.26, *P* = 0.61). They efficiently performed the task, making fewer errors during the training days (total and re-entry errors). We found a significant difference between adult male offspring of GD rats and the control group in the number of re-entry errors (*F* (7,77) = 2.61, *P* = 0.017, *d* = 1.695), and a paired test revealed that a higher number of re-entry errors were committed by GD group rats on days 7 and 8 (*t* = 2.88, *P* = 0.0086, *d* = 1.177 and *t* = 3.11, *P* = 0.0057, *d* = 1.695, respectively). These results show a short-term memory deficiency. 

Female GD offspring showed a significant decrease in the number of total errors in adulthood from days 3 to day 5 (*F* (7,63) = 2.15, *P* = 0.050) due to the reduction of the number of omissions, since the number of re-entry errors did not show significant differences compared with the control, and they efficiently performed the task during the training days.

### 3.6. Effects of GD on Oxidative Stress Biomarkers and ROS Production in Hippocampus and Cortex of First-Generation Offspring

To determine the effect of GD in oxidative stress on the cerebral cortex and hippocampus from both male and female offspring in young and adult rats, we first evaluated ROS production measured by 2′,7′-dichlorodihydrofluorescein diacetate probe. We found a significant increase in ROS production in male and female GD offspring, as shown in Table 3. In young male GD offspring, we found a ~31% increase in ROS production in the cerebral cortex compared with their respective control (t = 2.61, P = 0.0009, d = 1.26), with a ~66% increase in the hippocampus (t = 3.85, P = 0.0009, d = 1.64). Next, in the cerebral cortex of adult male GD offspring, a ~36% increase in ROS production was observed (t = 2.69, P = 0.017, d = 1.34). We also found a ~ 40% increase in the hippocampus of adult male GD offspring (t = 3.8, P = 0.0013, d = 1.70). Female GD offspring exhibited similar results in both the cerebral cortex (~22%, t = 2.45, P = 0.028, d = 1.27; ~ 41%, t = 2.87, P = 0.011, d = 1.39 young and adult rats, respectively) and the hippocampus (~36% t = 2.79, P = 0.015, d = 1.44; ~42% t = 3.50, P = 0.047, d = 1.75 young and adult rats, respectively). We also evaluated lipid-peroxidation using thiobarbituric acid-reactive substances (TBARS) in both the cerebral cortex and hippocampus in male and female GD offspring, and we found a significant increase in lipid-peroxidation as shown in Table 3. 

### 3.7. Effects of GD on the Antioxidant Enzymes Activities in First-Generation Offspring

Next, we evaluated catalase and SOD activities in the hippocampus and cerebral cortex of male and female rats of both ages. A significant decrease in the cerebral cortex catalase activity of young and adult male GD offspring rats was found (~43% and ~35%, respectively; Table 3). In the hippocampus, a decrease in catalase activity (~42% less) was observed only in adult male offspring compared with the control group (Table 3). The cerebral cortex and hippocampus of the female offspring of GD rats showed a decrease in catalase activity at young and adult ages (~48% and ~44% less in the cerebral cortex and ~44% and 50% reduced in the hippocampus, respectively) compared with their respective control (Table 3).

We evaluated SOD activity, and an activity decrease at both ages and in both structures was observed only in the female offspring of rats with gestational diabetes. No changes in the male offspring were observed (Table 3).

### 3.8. Effects of GD on the Glutathione Status of First-Generation Offspring

The glutathione status in the cortex and hippocampus was evaluated (Table 3). A reduced level of total glutathione in the cerebral cortex, the highest level of oxidized glutathione, and a decrease in reduced glutathione levels were found in young GD male offspring as well as a low GSH/GSSG ratio. The cerebral cortex of adult male offspring did not show statistical differences. In the hippocampus, an increase in total glutathione (~90%) was observed in the juvenile offspring of rats with gestational diabetes, without changes in the oxidized glutathione, reduced glutathione levels and GSH/GSSG ratio. No significant changes in the glutathione status were found in the cerebral cortex of young GD female offspring. However, in the hippocampus of young GD female offspring, we found a decrease in reduced glutathione and a minor GSH/GSSG ratio. Adult GD female offspring exhibit a decrease in total glutathione levels with an increase in oxidized glutathione and a decrease in reduced glutathione levels in the cerebral cortex as well as the lowest GSH/GSSG ratio. Finally, a decrease in reduced glutathione in the hippocampus of adult GD offspring was found. 

### 3.9. Effects of GD on the Biochemical Serum Parameters of First-Generation Offspring

To determine the impact of gestational diabetes on metabolic parameters, the glucose, insulin, cholesterol, and triglycerides in the serum of young and adult male and female offspring were measured (Table 4). No statistical differences were observed in the glucose and total cholesterol of male or female, and young or adult GD offspring. The triglycerides level was higher in young and adult male GD offspring (*t* = 2.243, *P* = 0.041, *d* = 1.13, and *t* = 2.341, *P* = 0.037, *d* = 1.25, respectively), whereas the female GD offspring showed a higher level in this parameter only in adulthood (*t* = 2.256, *P* = 0.0319, *d* = 1.366). The HDL−cholesterol level was low in young and adult male GD offspring (*t* = 3.48, *P* = 0.003, *d* = 1.64, and *t* = 23.87, *P* = 0.001, respectively), and the insulin serum level was low in young male GD offspring (*t* = 2.33, *P* = 0.025, *d* = 1.88). This change did not remain into adulthood. Similar to young GD offspring, we observed a low level of HDL−cholesterol in young and adult female offspring (*t* = 4.83, *P* = 0.0002, *d* = 2.34, and *t* = 4.40, *P* = 0.0006, *d* = 2.20, respectively) and a higher insulin level at both ages (*t* = 2.37, *P* = 0.032, *d* = 1.18, and *t* = 2.47, *P* = 0.023, *d* = 1.107, respectively).

### 3.10. Correlation Between Behavior Parameters and Oxidative Stress

Finally, we calculated the Pearson correlation coefficients between the parameters of the behavioral tests and oxidative stress biomarkers. Only correlations that were significant at *P* < 0.05 were considered, as shown in Table 5. In anxiety behavior, we found a significant correlation between the time in closed arms and SOD activity in hippocampus and cerebral cortex in young male and adult female rats, indicating that the more time spent in the closed arms of the elevated plus maze, the greater the SOD activity. In adult females, a significant correlation of this behavior with the enzyme catalase was also found. We compared the open field behavior and we found a correlation between time in the open area and the production of ROS in the hippocampus of young male rats, suggesting an increase in the production of ROS in the hippocampus promotes reduced anxiety-like behavior. In adult females’ rats, we found a significant inverse correlation between the time spent in the open area and the SOD and catalase activities in the cerebral cortex. Therefore, reduced anxiety-like behavior is correlated with lower activity of these enzymes in the cerebral cortex. In the Morris water maze studies, we found a correlation between the average distance traveled during the eight training days and ROS production in cerebral cortex of young males, indicating a relationship between in the increase in ROS and the distance traveled to find the platform. In young female rats, we found a significant inverse correlation of the distance traveled with reduced SOD activity in both the hippocampus and cerebral cortex. This finding indicates a potential relationship between oxidative stress and behavior abnormalities in offspring of GD rats. We did not find a correlation between the number of re−entry errors with respect to oxidative stress biomarkers (data not shown).

## 4. Discussion

In this study, we examined the effects of gestational diabetes on oxidative stress and their relationship with behavioral impairment linked to the hippocampus and cerebral cortex function in young and adult rat offspring. Adverse environment exposure during embryonic and early postnatal development may lead to a risk of developing metabolic disease in later life [7,31,32,33]. 

Gestational diabetes modifies the offspring’s body weight at birth in a maternal glucose-dependent manner. Our results only indicated a lower body weight at birth (microsomia) in male offspring of GD rats, and this change remained during the weaning, juvenile, and adult stages. This weight reduction at birth could be due to the high blood glucose concentration in the intrauterine milieu during gestational diabetes, since it was reported to induce pancreatic hypertrophy due to the over-stimulation of pancreatic β cells, preventing the secretion of insulin and decreasing anabolism and, subsequently, neonatal microsomia [3,34,35,36], this results are according with [37]. The female offspring of GD showed a lower body weight only during weaning, without changes at birth or juvenile and adult stages. These results suggest that the male offspring of GD rats are more susceptible to disturbances in body weight, when they are exposed to a high maternal blood glucose level during the middle stage of pregnancy. In this regard, sexual differences in the offspring body weight have been reported in relation to maternal obesity and a maternal high-fat diet model [38,39], where it was shown that the body weight of female offspring did not change. 

Our most striking findings are the cognitive changes in the offspring exposed to gestational diabetes. Several studies have reported disturbances in the anxiety-like behavior of gestational diabetes offspring. Ramanathan et al. [40] showed that maternal diabetes offspring displayed hyperactivity in the open field test and anxiety in the elevated plus maze at two months of age (young rats). We found a lower level of anxiety-like behavior in the male offspring of GD rats at the juvenile age, which remained into adulthood, whereas in adult female offspring, similar to adult male offspring, a low level of anxiety-like behavior was observed. These results agree with those reported by Chandna et al. [41], who observed the same behavior in the male offspring of rats with gestational diabetes at the postnatal day 58 (young). The discrepancy between our results and those of Ramanathan et al. [40] could be due to differences in the gestational diabetes model and STZ dose. Ramanathan et al. used a dose of 50 mg/kg of body weight and employed a pregestational model. In our experiments, as well as in Chandna et al.’s model [41], a low dose of STZ in the middle stage of pregnancy (38 mg/kg body weight) was used. These observations indicate that the behavioral abnormalities in the offspring of diabetic rats are dependent on the level of intrauterine hyperglycemia, as well the time of exposure. 

Currently, few studies have assessed the effect of gestational diabetes on cognitive performance. In this study, we evaluated spatial learning and short-term memory. We obtained findings of age- and sex-dependency. Whereas the male offspring of gestational diabetic rats at young and adult ages did not show disruptions in spatial learning, the female progeny showed an impairment of cognitive performance at the juvenile age. These results are in accordance with those reported by Kinney et al. [42], who found learning deficits, determined through the use of the Lashley III maze, only in female offspring at 60 days of age. In our work, female offspring traveled a greater distance to reach the escape platform during the training days in the Morris water maze. However, this deficit did not remain into adulthood. Estrogens have been reported to exert protective effects on neuronal cells and may contribute to the normal process of memory consolidation. Thus, the deficit reversion observed in adult female rats could be due to the effect of estrogens [43,44]. In addition, disturbances in spatial learning and deficiencies in working memory (a cortex-dependent task) have been described in metabolic disorders, such as type I and II diabetes mellitus, as well as in over-nourished offspring [45] and during the early post-natal stage of offspring of gestational diabetes rats [46], which was linked to a modification of the neurotransmitter levels and cell death. Our results show that the adult female offspring of diabetic rats showed minor total errors at days 3–5 of training, compared with control rats, which was due to a reduced number of error omissions committed by the diabetic offspring rats, which is possibly related to the lower anxiety level presented in this group. However, their re-entry errors were similar to those of the control group, implying that no differences in working memory existed in diabetic female offspring rats of any age. In male offspring, we observed a deficit in working memory only at the adult age, at which the rats committed a higher number of re-entry errors. This result differs from that reported by Kim et al. [46] (short-term memory impairment), which could be due to their use of a different task. They realized an avoidance task, in which a possible alteration to the amygdala could strongly influence the performance. They showed an increase in hippocampal apoptosis, a brain area related to long-term memory or spatial information. Conversely, we used the radial maze, which is a task that implies hippocampal–prefrontal cortex communication dependence. 

Optimal brain function is dependent on cellular redox homeostasis, and neurodegenerative diseases, cognitive decline, and cerebral dysfunction are closely related to oxidative stress. At the embryonic stage, under hyperglycemia, a link with oxidative stress has been reported in the intrauterine milieu, and ROS reduction contributes to the decrease in oxidative damage to developing embryos in rats with gestational diabetes [19,47]. For the first time, the influence of gestational diabetes on oxidative stress in specific brain areas, the cerebral cortex and hippocampus, was examined. We found a significant increase in reactive ROS using H2DCFDA. To corroborate if this increase in oxidants was related with oxidative damage in lipids, we also evaluated lipid peroxidation and we found a consistent increase. The brain is highly susceptible to lipid peroxidation due a high polyunsaturated lipids content, high oxygen consumption, and a relativity poor antioxidant defense [21]. 4-Hydroxynonenal (4-HNE) and malondialdehyde (MDA) are two highly reactive aldehydes, by-products of lipid peroxidation implicated in the pathogenesis of a variety of neuropsychiatric disorders, for example, Alzheimer's disease, depression, Parkinson's disease, and schizophrenia [48]. The oxidation of polyunsaturated lipids in the cell membrane leads to significant changes in cell integrity and in membrane permeability, affecting various transmembrane processes, such as receptors activation, formation of intracellular second messenger and Ca^2+^ homeostasis, and impairment of glutamate transport and mitochondrial function in synapses [49]. 

These results suggest that the cerebral cortex and hippocampus are susceptible to oxidative damage and are therefore liable to present an altered function. The antioxidant system was disturbed in male and female GD offspring in the cerebral cortex and hippocampus at the juvenile age, which remained into adulthood. Oxidative stress resulting from a hyperglycemic intrauterine milieu increased the oxidative damage and alteration in the antioxidant defense during fetal [18] and neonatal [17] stages in the peripheric organs (the liver, kidney, and pancreas). Our results showed for the first time that these changes during the fetal stage remain into adulthood and specifically in the cerebral cortex and hippocampus. The increases in ROS, SOD, and catalase activities are correlated with behavior abnormalities in the offspring of GD dams. Oxidative stress could modify the normal brain development of GD offspring and lead to cognitive deficiency. We think that a more complex relationship exists between oxidative stress and cognitive function due to the reactive oxygen/nitrogen species, which act as signals in the regulation of many processes in neuronal development and brain function [19].

According to many studies that support the link between exposure to an adverse intrauterine milieu, including hyperglycemia, and a subsequent risk of developing metabolic diseases [3,9,50,51,52], our results show a metabolic disruption in male and female GD offspring. The male progeny only showed modifications in lipid metabolism in adulthood, whereas in female offspring, we observed an increase in the insulin level, indicative of insulin resistance, and a lipid metabolism alteration at both ages, indicating a sex-dependent metabolic disruption. Hence, GD, during the middle stage of gestation with a moderate glucose level in the intrauterine milieu, is enough to permanently modify the health of young rats.

In summary, these results suggest that gestational diabetes modifies oxidative stress and cognitive behavior in an age- and sex-dependent manner. However, the relationship between oxidative stress and cognitive performance is complex. Therefore, the evaluation of other possible mediators, such as the impact of oxidative stress and the inflammatory response to neuronal function and neuronal morphology, is necessary to elucidate the possible underlying mechanisms associated with behavioral/cognitive alterations and their possible effect across generations.

## 5. Conclusions 

Gestational diabetes modifies the oxidative stress in the cerebral cortex and hippocampus as well as cognitive behavior in an age- and sex-dependent manner.

## Figures and Tables

**Table 1:** Glucose levels and body weight of control and diabetic dams during the pregnancy.

		GC	GD
Fasting blood glucose (mg/dL)	Pre-induction (day 9)	88.5 ± 4.1	93.17 ± 4.9
	Post-induction (day 11)	77.0 ± 2.0	246.8 ± 48.7 *
Postprandial blood glucose (mg/dL)	Day 12	76.0 ± 2.0	423.0 ± 25.8 *
	Day 16	87.0 ± 6.8	398.8 ± 46.5 *
	Day 20	88.3 ± 5.2	296.8 ± 49.8 *
Body weight (g)	Day 12	333.3 ± 13.3	338.0 ± 18.0
	Day 16	381.7 ± 2.8	378.0 ± 6.9
	Day 20	420.0 ± 8.1	404.0 ± 13.8

Note: Results are expressed as the mean ± standard error of the mean (SEM). Student’s t-test; * *p* < 0.05. GC, pregnant control rats (n = 6); GD, rats with gestational diabetes (n = 6).

**Table 2:** Offspring body weight at different ages.

Body Weight (g)								
Age	Male Offspring		*n*		Female Offspring		*n*	
	**C**	**GD**	**C**	**GD**	**C**	**GD**	**C**	**GD**
Birth	6.82 ± 0.16	5.81 ± 0.14 *^●^	28	34	6.35 ± 0.24	5.99 ± 0.22	20	30
Weaning (21 days)	37.3 ± 0.93	24.86 ± 1.06 *^●^	28	34	36.41 ± 1.36	26.71 ± 1.07 *^●^	20	30
Young (2 months)	295.2 ± 10.85	261.5 ± 14.26 *^●^	10	10	204.0 ± 3.84	196.0 ± 4.11	10	10
Adult (6 months)	514.4 ± 14.59	436.8 ± 20.8 *^●^	12	12	281.4 ± 16.14	282.5 ± 5.60	10	10

Note: Data are expressed as the mean ± SEM. Student’s t-test; * *p* < 0.05; ^●^ d > 1.0. C: control offspring, GD: gestational diabetes offspring.

**Table 3:** Oxidative stress biomarkers. Effect of gestational diabetes in oxidative stress biomarkers of cerebral cortex and hippocampus of young and adult, male and female offspring.

	**Male Offspring**				**Female Offspring**			
**Cerebral Cortex**	**C-Young** ***n = 6***	**GD-Young** ***n = 6***	**C-Adult** ***n = 6***	**GD-Adult** ***n = 6***	**C-Young** ***n = 5***	**GD-Young** ***n = 5***	**C-Adult** ***n = 5***	**GD-Adult** ***n = 5***
GSHt (µmoles/mg protein)	0.39 ± 0.04	0.22 ± 0.04 *^●^	0.36 ± 0.04	0.42 ± 0.01	0.38 ± 0.1	0.32 ± 0.02	0.69 ± 0.03	0.49 ± 0.04 *^●^
GSSG (µmoles/mg protein)	0.01 ± 0.001	0.13 ± 0.02 *^●^	0.10 ± 0.01	0.15 ± 0.016	0.17 ± 0.02	0.18 ± 0.03	0.08 ± 0.01	0.26 ±0.04 *^●^
GSH (µmoles/mg protein)	0.37 ± 0.04	0.11 ± 0.03 *^●^	0.25 ± 0.05	0.27 ± 0.005	0.21 ± 0.02	0.13 ± 0.04	0.61 ± 0.03	0.23 ± 0.07 *^●^
GSH/GSSG ratio	14.02 ± 2.16	1.14 ± 0.33 *^●^	2.66 ± 0.68	1.75 ± 0.18	1.36 ± 0.35	0.84 ± 0.37	8.44 ± 1.73	0.97 ± 0.38 *^●^
ROS (arbitrary units/mg protein)	27.39 ± 2.56	36.1 ± 2.53 *^●^	21.85 ± 2.15	29.74 ± 1.98 *^●^	28.52 ± 0.97	34.81 ± 2.23 *^●^	28.27 ± 3.01	41.19 ± 3.33 *^●^
Lipid peroxidation (nmoles TBARS/mg protein)	330.0 ± 18.21	412.9 ± 29.1 *^●^	241.1 ± 15.82	329.0 ± 33.18 *^●^	328.2 ± 10.14	433.7 ± 18.68 *^●^	309.7 ± 27.23	382.3 ± 17.91 *^●^
Catalase activity (U/mg protein)	1.58 ± 0.17	0.87 ± 0.06 *	1.24 ± 0.08	0.79 ± 0.11 *	2.00 ± 0.17	1.03 ± 0.12 *^●^	1.88 ± 0.19	1.05 ± 0.100 *^●^
SOD activity (U/mg protein)	2.96 ± 0.15	2.97 ± 0.017	1.59 ± 0.166	1.90 ± 0.13	12.76 ± 0.62	8.18 ± 0.44 *^●^	7.95 ± 0.38	2.57 ± 0.085 *^●^
**Hippocampus**	**C-Young** ***n = 6***	**GD-Young** ***n = 6***	**C-Adult** ***n = 6***	**GD-Adult** ***n = 6***	**C-Young** ***n = 5***	**GD-Young** ***n = 5***	**C-Adult** ***n = 5***	**GD-Adult** ***n = 5***
GSHt (µmoles/mg protein)	0.21 ± 0.02	0.41 ± 0.02 *^●^	0.28 ± 0.03	0.38 ± 0.06	0.42 ± 0.00	0.18 ± 0.03	0.68 ± 0.0	0.47 ± 0.02
GSSG (µmoles/mg protein)	0.07 ± 0.004	0.13 ± 0.03	0.15 ± 0.02	0.17 ± 0.02	0.053 ± 0.00	0.06± 0.00	0.081 ± 0.0	0.05 ± 0.0
GSH (µmoles/mg protein)	0.14 ± 0.03	0.28 ± 0.02	0.13 ± 0.01	0.21 ± 0.04	0.36 ± 0.01	0.12 ± 0.04 *^●^	0.60 ± 0.01	0.41 ± 0.01 *^●^
GSH/GSSG ratio	2.10 ± 0.55	2.33 ± 0.67	0.92 ± 0.24	1.22 ± 0.33	7.01 ± 0.8	2.05 ± 0.96 *^●^	8.17 ± 1.84	8.60 ± 1.6
ROS (arbitrary units/mg protein)	17.98 ± 1.24	29.9 ± 2.61 *^●^	17.08 ± 1.74	24.03 ± 0.84 *^●^	17.97 ± 2.18	24.46 ± 1.03 *	17.8 ± 2.90	25.44 ± 1.96 *^●^
Lipid peroxidation (nmoles TBARS/mg protein)	348.6 ± 18.15	403.0 ± 11.85 *^●^	197.7 ± 15.97	320.7 ± 33.01 *^●^	276.5 ± 18.8	393.6 ± 35.93 *^●^	288.2 ± 17.75	420.3 ± 45.54 *^●^
Catalase activity (U/mg protein)	1.15 ± 0.17	0.74 ± 0.08	1.41 ± 0.09	0.80 ± 0.04 *^●^	1.45 ± 0.16	0.81 ± 0.14 *^●^	1.33 ± 0.09	0.66 ± 0.09 *^●^
SOD activity (U/mg protein)	7.02 ± 0.23	6.95 ± 0.20	5.43 ± 0.21	4.87 ± 0.02	12.76 ± 0.62	8.12 ± 0.44 *^●^	7.72 ± 0.56	3.27 ± 0.04 *^●^

Note: C, control offspring. GD, gestational diabetes offspring (n = 5–6). GSHt, total glutathione. GSSG, oxidized glutathione. GSH, reduced glutathione. ROS, reactive oxygen species. TBARS, thiobarbituric acid-reactive substances. SOD, superoxide dismutase. Data are expressed as mean ± SEM. Student’s t-test; * *P* < 0.05, ^●^ d > 1.

**Table 4:** Metabolic parameters of offspring.

**Male Offspring**				
	**C-Young** ***n* = 8**	**GD-Young** ***n* = 8**	**C-Adult** ***n* = 7**	**GD-Adult** ***n* = 7**
Glucose (mg/dL)	90.27 ± 2.07	85.4 ± 3.03	91.92 ± 1.63	91.35 ± 3.31
Insulin (ng/mL)	0.42 ± 0.027	0.33 ± 0.015 *	0.44 ± 0.023	0.42 ± 0.029
Cholesterol (mg/dL)	71.84 ± 1.92	75.89 ± 2.71	73.43 ± 2.18	68.2 ± 1.98
Triglycerides (mg/dL)	33.3 ± 2.84	43.46 ± 3.31 *	40.24 ± 2.55	53.94 ± 5.26 *
HDL-cholesterol (mg/dL)	52.72 ± 1.95	40.1 ± 3.05 *	50.63 ± 3.04	38.1 ± 1.43 *
**Female Offspring**				
	**C-Young** ***n* = 7**	**GD-Young** ***n* = 10**	**C-Adult** ***n* = 9**	**GD-Adult** ***n* = 7**
Glucose (mg/dL)	82.22 ± 3.28	82.69 ± 2.49	86.44 ± 2.23	87.63 ± 2.01
Insulin (ng/mL)	0.32 ± 0.002	0.36 ± 0.014 *	0.32 ± 0.004	0.48 ± 0.064 *
Cholesterol (mg/dL)	85.63 ± 1.61	85.44 ± 1.72	74.29 ± 2.28	80.61 ± 4.8
Triglycerides (mg/dL)	37.96 ± 1.87	43.04 ± 2.46	37.74 ± 6.46	54.25 ± 2.89 *
HDL-cholesterol (mg/dL)	62.46 ± 1.60	49.06 ± 2.18 *	51.38 ± 3.04	34.73 ± 2.24 *

Note: Data are expressed as the mean ± SEM. Student’s t-test; * *p* < 0.05.

**Table 5:** Pearson correlation between behavior parameters and oxidative stress biomarkers.

	Male Offspring						Female offspring					
	Young			Adult			Young			Adult		
**Cerebral cortex**	**ROS**	**SOD**	**CAT**	**ROS**	**SOD**	**CAT**	**ROS**	**SOD**	**CAT**	**ROS**	**SOD**	**CAT**
Time in closed arms** (Elevated plus maze)	-	*r* = 0.709 *P* = 0.009	-	*r* = −0.660 *P* = 0.019	-	-	-	-	-	-	*r* = 0.614 *P* = 0.033	-
Time in open area** (Open field)	-	-	-	-	-	-	-	-	-	-	*r* = −0.651 *P* = 0.021	*r* = −0.793 *P* = 0.002
Distance traveled** (Morris water maze)	*r* = 0.746 *P* = 0.005	-	-	-	-	-	-	*r* = −0.587 *P* = 0.048	-	-	-	
**Hippocampus**	**ROS**	**SOD**	**CAT**	**ROS**	**SOD**	**CAT**	**ROS**	**SOD**	**CAT**	**ROS**	**SOD**	**CAT**
Time in closed arms** (Elevated plus maze)	-	*r* = 0.815 *P* = 0.001	-	-	-	-	-	-	-	-	-	*r* = 0.741 *P* = 0.005
Time in open area** (Open field)	*r* = 0.601 *P* = 0.038	-	-	-	-	-	-	-	-	-	-	-
Distance traveled** (Morris water maze)	-	-	-	-	-	-	--	*r* = −0.577 *P* = 0.049	-	-	-	-

Note: Pearson correlation coefficient (r) between parameters of the behavioral tests and oxidative stress biomarkers; ROS, reactive oxygen species; SOD, superoxide dismutase; CAT, catalase; young male, n = 6; adult male, n = 6; young female, n = 5; adult female n = 5.

## References

[1] WHO Reproductive Health Library WHO recommendation on the diagnosis of gestational diabetes in pregnancy. https://extranet.who.int/rhl/topics/preconception-pregnancy-childbirth-and-postpartum-care/antenatal-care/who-recommendation-diagnosis-gestational-diabetes-pregnancy-0.

[2] International Diabetes Federation (2017). IDF Diabetes Atlas.

[3] Aerts L., Van Assche F.A. (2006). Animal evidence for the transgenerational development of diabetes mellitus. Int. J. Biochem. Cell Biol..

[4] Seki Y., Williams L., Vuguin P.M., Charron M.J. (2012). Minireview: Epigenetic programming of diabetes and obesity: Animal models. Endocrinology.

[5] Liu H.W., Mahmood S., Srinivasan M., Smiraglia D.J., Patel M.S. (2013). Developmental programming in skeletal muscle in response to over nourishment in the immediate postnatal life in rats. J. Nutr. Biochem..

[6] Friedman J.E. (2015). Obesity and gestational diabetes mellitus pathways for programming in mouse, monkey, and man—where do we go next? The 2014 Norbert Freinkel Award Lecture. Diabetes Care..

[7] Rodrıguez R.R., Renauld A., Celener D., Pérez R.L., Susemihl M.C. (1998). Offspring of streptozotocin diabetic rats: Size changes in Langerhans islets with time after birth. Diabetes Res. Clin. Prac..

[8] Catalano P.M., Thomas A., Huston-Presley L., Amini S.B. (2003). Increased fetal adiposity: A very sensitive marker of abnormal in utero development. Am. J. Obstet. Gynecol..

[9] Blondeau B., Joly B., Perret C., Prince S., Bruneval P., Lelièvre-Pégorier M., Van Huyen J.P.D. (2011). Exposure in utero to maternal diabetes leads to glucose intolerance and high blood pressure with no major effects on lipid metabolism. Diabetes Metab..

[10] Dalley J.W., Cardinal R.N., Robbins T.W. (2004). Prefrontal executive and cognitive functions in rodents: Neural and neurochemical substrates. Neurosci. Biobehav. Rev..

[11] DeBoer T., Wewerka S., Bauer P.J., Georgieff M.K., Nelson C.A. (2005). Explicit memory performance in infants of diabetic mothers at 1 year of age. Dev. Med. Child Neurol..

[12] Cai S., Qiu A., Broekman B.F., Wong E.Q., Gluckman P.D., Godfrey K.M., Saw M.S., Soh S.E., Kwek K., Chong K. (2016). The influence of gestational diabetes on neurodevelopment of children in the first two years of life: A prospective study. PLoS ONE.

[13] Ornoy A., Ratzon N., Greenbaum C., Wolf A., Dulitzky M. (2001). School-age children born to diabetic mothers and to mothers with gestational diabetes exhibit a high rate of inattention and fine and gross motor impairment. J. Pediatric Endocrinol. Metab..

[14] Jing Y.H., Song Y.F., Yao Y.M., Yin J., Wang D.G., Gao L.P. (2014). Retardation of fetal dendritic development induced by gestational hyperglycemia is associated with brain insulin/IGF-I signals. Int. J. Dev. Neurosci..

[15] Wrigley S., Arafa D., Tropea D. (2017). Insulin-like growth factor 1: At the crossroads of brain development and aging. Front Cell Neurosci..

[16] Golalipour M.J., Kafshgiri S.K., Ghafari S. (2012). Gestational diabetes induced neuronal loss in CA1 and CA3 subfields of rat hippocampus in early postnatal life. Folia Morphol..

[17] Raza H., John A. (2004). Glutathione metabolism and oxidative stress in neonatal rat tissues from streptozotocin-induced diabetic mothers. Diabetes Metab. Res. Rev..

[18] Kamel M.A., Helmy M.H., Hanafi M.Y., Mahmoud S.A., Elfetooh H.A. (2014). Effect of maternal diabetes on pre-and post-natal redox status of F1 rat offspring. Open J. Endocr. Metab. Dis..

[19] Wang X., Michaelis E.K. (2010). Selective neuronal vulnerability to oxidative stress in the brain. Front Aging Neurosci..

[20] Liu Z., Zhou T., Ziegler A.C., Dimitrion P., Zuo (2017). Oxidative stress in neurodegenerative diseases: From molecular mechanisms to clinical applications. Oxid. Med. Cell Longev..

[21] Rego A.C., Oliveira C.R. (2003). Mitochondrial dysfunction and reactive oxygen species in excitotoxicity and apoptosis: Implications for the pathogenesis of neurodegenerative diseases. Neurochem. Res..

[22] Alvarez E.O., Beauquis J., Revsin Y., Banzan A.M., Roig P., De Nicola A.F., Saravia F. (2009). Cognitive dysfunction and hippocampal changes in experimental type 1 diabetes. Behav. Brain. Res..

[23] Suge R., Shimazu T., Hasegawa H., Inoue I., Hayashibe H., Nagasaka H., Watanabe S.I. (2012). Cerebral antioxidant enzyme increase associated with learning deficit in type 2 diabetes rats. Brain Res..

[24] Rebolledo-Solleiro D., Roldán-Roldán G., Díaz D., Velasco M., Larqué C., Rico-Rosillo G., Vega-Robledo G., Zambrano E., Hiriart M., Pérez de la Mora M. (2017). Increased anxiety-like behavior is associated with the metabolic syndrome in non-stressed rats. PLoS ONE.

[25] Zemdegs J., Quesseveur G., Jarriault D., Pénicaud L., Fioramonti X., Guiard B.P. (2016). High-fat diet-induced metabolic disorders impairs 5-HT function and anxiety-like behavior in mice. British J. Pharmacol..

[26] Gornall A.G., Bardawill C.J., David M.M. (1949). Determination of serum proteins by means of the biuret reaction. J. Biol. Chem..

[27] Buege J.A., Aust S.D. (1978). Microsomal lipid peroxidation. Methods Enzymol..

[28] Ortiz-Avila O., Sámano-García C.A., Calderón-Cortés E., Pérez-Hernández I.H., Mejía-Zepeda R., Rodríguez-Orozco A.R., Saavedra-Molina A., Cortés-Rojo C. (2013). Dietary avocado oil supplementation attenuates the alterations induced by type I diabetes and oxidative stress in electron transfer at the complex II-complex III segment of the electron transport chain in rat kidney mitochondria. J. Bioenerg. Biomemb..

[29] Akerboom T.P., Sies H. (1981). Assay of glutathione, glutathione disulfide, and glutathione mixed disulfides in biological samples. Methods Enzymol..

[30] Jeulin C., Soufir J.C., Weber P., Laval-Martin D., Calvayrac R. (1989). Catalase activity in human spermatozoa and seminal plasma. Gamete. Res..

[31] Smith N.H., Ozanne S.E. (2006). Intrauterine origins of metabolic disease. Rev. Gynecol. Peri. Pract..

[32] Vickers M. (2014). Early life nutrition, epigenetics and programming of later life disease. Nutrients.

[33] Han J., Xu J., Long Y.S., Epstein P.N., Liu Y.Q. (2007). Rat maternal diabetes impairs pancreatic β-cell function in the offspring. Am. J. Physiol. Endocrinol. Metab..

[34] Aerts L., Holemans K., Van Assche F.A. (1990). Maternal diabetes during pregnancy: Consequences for the offspring. Diabetes Metab. Rev..

[35] López-Soldado I., Herrera E. (2003). Different diabetogenic response to moderate doses of streptozotocin in pregnant rats, and its long-term consequences in the offspring. J. Diabetes Res..

[36] Fetita L.S., Sobngwi E., Serradas P., Calvo F., Gautier J.F. (2006). Consequences of fetal exposure to maternal diabetes in offspring. J. Clin. Endocrinol. Metab..

[37] Piazza F.V., Segabinazi E., de Meireles A.L.F., Mega F., de Figueiredo Spindler C., Augustin O.A., Salvalaggio G.D.S., Achaval M., Kruse M.S., Coirini H. (2019). Severe Uncontrolled Maternal Hyperglycemia Induces Microsomia and Neurodevelopment Delay Accompanied by Apoptosis, Cellular Survival, and Neuroinflammatory Deregulation in Rat Offspring Hippocampus. Cell. Mol. Neurobiol..

[38] Panchenko P.E., Lacroix M.C., Jouin M., Voisin S., Badonnel K., Lemaire M., Durieux D. (2019). Effect of Maternal Obesity and Preconceptional Weight Loss on Male and Female Offspring Metabolism and Olfactory Performance in Mice. Nutrients.

[39] Wankhade U.D., Zhong Y., Kang P., Alfaro M., Chintapalli S.V., Piccolo B.D., Shankar K. (2018). Maternal high-fat diet programs offspring liver steatosis in a sexually dimorphic manner in association with changes in gut microbial ecology in mice. Sci. Rep..

[40] Ramanathan M., Jaiswal A.K., Bhattacharya S.K. (2000). Hyperglycaemia in pregnancy: Effects on the offspring behavior with special reference to anxiety paradigms. Indian J. Exp. Biol..

[41] Chandna A.R., Kuhlmann N., Bryce C.A., Greba Q., Campanucci V.A., Howland J.G. (2015). Chronic maternal hyperglycemia induced during mid-pregnancy in rats increases RAGE expression, augments hippocampal excitability, and alters behavior of the offspring. Neuroscience.

[42] Kinney B.A., Rabe M.B., Jensen R.A., Steger R.W. (2003). Maternal hyperglycemia leads to gender-dependent deficits in learning and memory in offspring. Exp. Biol. Med..

[43] McEwen B.S., Alves S.E. (1999). Estrogen actions in the central nervous system. Endocr. Rev..

[44] Luine V.N. (2014). Estradiol and cognitive function: Past, present and future. Horm. Behav..

[45] Sarker G., Peleg-Raibstein D. (2019). Maternal overnutrition induces long-term cognitive deficits across several generations. Nutrients.

[46] Kim Y.H., Sung Y.H., Lee H.H., Ko I.G., Kim S.E., Shin M.S., Kim B.K. (2014). Postnatal treadmill exercise alleviates short-term memory impairment by enhancing cell proliferation and suppressing apoptosis in the hippocampus of rat pups born to diabetic rats. J. Exerc. Rehabil..

[47] Dong D., Yu J., Wu Y., Fu N., Villela N.A., Yang P. (2015). Maternal diabetes triggers DNA damage and DNA damage response in neurulation stage embryos through oxidative stress. Biochem. Biophys. Res. Commun..

[48] Romano A., Serviddio G., Calcagnini S., Villani R., Giudetti A.M., Cassano T., Gaetani S. (2017). Linking lipid peroxidation and neuropsychiatric disorders: Focus on 4-hydroxy-2-nonenal. Free Radic. Biol. Med..

[49] Keller J.N., Mark R.J., Bruce A.J., Blanc E., Rothstein J.D., Uchida K., Waeg G., Mattson P. (1997). 4–Hydroxynonenal, an aldehydic product of membrane lipid peroxidation, impairs glutamate transport and mitochondrial function in synaptosomes. Neuroscience.

[50] Oswald M.C., Garnham N., Sweeney S.T., Landgraf M. (2018). Regulation of neuronal development and function by ROS. FEBS lett..

[51] Poston L. (2011). Intergenerational transmission of insulin resistance and type 2 diabetes. Prog. Biophys. Mol. Biol..

[52] Pinney S.E., Simmons R.A. (2012). Metabolic programming, epigenetics, and gestational diabetes mellitus. Curr. Diabetes Rep..

